# Supplementary material for: Arbuscular Mycorrhizal Fungal Communities of Native Plant Species under High Petroleum Hydrocarbon Contamination Highlights Rhizophagus as a Key Tolerant Genus
Source: Microorganisms. 2020 Jun 9;8(6):872. doi: 10.3390/microorganisms8060872 (PMC7356029; doi:10.3390/microorganisms8060872)
Supplement: Supplementary file 1 [file microorganisms-08-00872-s001.pdf]

Supplementary material

Table S1. The concentration of different petroleum hydrocarbon pollutants recorded in the three sampled basins compared with the reference values set by Quebec Government.

Petroleum hydrocarbon	Concentration recorded in the different basins µg/kg			Threshold values set by the Quebec Government (ccme.ca)		
	LC[1]	MC	HC	Residential	Commercial	Industrial
Phenanthrene	1	2700	4300	0.1	5	50
Acenaphrene	0.2	760	620	0.1	10	100
Anthracene	6.7	340	570	0.1	10	100
Fluorene	0.3	710	630	0.1	10	100
1-methylnaphtalene	0.1	320	300	0.1	1	10
1,3-Dimethylnaphtalene	0.1	390	580	0.1	1	10
Total petroleum hydrocarbons (C10-C50)	3000	41000	91000	300	700	3500

[1] Low concentration (LC), moderate concentration (MC) and high concentration (HC).

Table S2. Identification of OTUs. BLAST results (using NCBI and MAARJAM database) of the 36 OTUs are given below. OTUs represented by two or less than two sequences (marked as red color) were not used for future analysis.

OTUs	Identification	*Orders*	*Families*	*Genera*	Accessing code	Similarity
OTU01	*Rhizophagus irregulare VTX00114*	*Glomerale*	*Glomeraceae*	*Rhizophagus*	FN600536.1	100%
OTU02	*Claroideoglomus* sp. *VTX00193*	*Glomerale*	*Claroideoglomeraceae*	*Claroideoglomus*	KJ809525.1	100%
OTU03	*Acaulospora* sp. *VTX00028*	*Diversisporale*	*Acaulosporacea*	*Acaulospora*	EU332727.1	99%
OTU04	*Rhizophagus* sp. *VTX00113*	*Glomerale*	*Glomeraceae*	*Rhizophagus*	JX144124.1	99%
OTU05	*Diversispora eburnea VTX00060*	*Diversisporale*	*Diversisporaceae*	*Diversispora*	AM713429.1	100%
OTU06	*Claroideoglomus* sp. *VTX00276*	*Glomerale*	*Claroideoglomeraceae*	*Claroideoglomus*	AB749515.1	99%
OTU07	*Paraglomus* sp. *VTX00350*	*Paraglomerale*	*Paraglomeraceae*	*Paraglomus*	HE576915.1	91%
OTU08	*Funneliformis mosseae VTX00067*	*Glomerale*	*Glomeraceae*	*Funneliformis*	JX461236.1	98%
OTU09	*Diversispora celata VTX00060*	*Diversisporale*	*Diversisporaceae*	*Diversispora*	AM713423.1	96%
OTU10	*Rhizophagus* sp. *VTX00114*	*Glomerale*	*Glomeraceae*	*Rhizophagus*	KC708370	98%
OTU11	*Glomeraceae* sp. *VTX00327*	*Glomerale*	*Glomeraceae*	-	JF414193	94%
OTU12	*Claroideoglomus* sp. *VTX00193*	*Glomerale*	*Claroideoglomeraceae*	*Claroideoglomus*	KJ809525.1	95%
OTU13	*Glomeraceae* sp. *VTX00069*	*Glomerale*	*Glomeraceae*	-	GU353706.1	96%
OTU14	*Glomeraceae* sp. *VTX00130*	*Glomerale*	*Glomeraceae*	-	AB698566.1	99%
OTU15	*Claroideoglomus* sp. *VTX00193*	*Glomerale*	*Claroideoglomeraceae*	*Claroideoglomus*	KJ809525.1	97%
OTU16	*Rhizophagus irregulare VTX00114*	*Glomerale*	*Glomeraceae*	*Rhizophagus*	FN600538.1	97%
OTU17	*Claroideoglomus* sp. *VTX00193*	*Glomerale*	*Claroideoglomeraceae*	*Claroideoglomus*	KJ809525.1	97%
OTU18	*Claroideoglomus* sp. *VTX00193*	*Glomerale*	*Claroideoglomeraceae*	*Claroideoglomus*	KJ809525.1	95%
OTU19	*Rhizophagus* sp.*VTX00113*	*Glomerale*	*Glomeraceae*	*Rhizophagus*	HG004504.1	96%
OTU20	*Rhizophagus* sp.*VTX00113*	*Glomerale*	*Glomeraceae*	*Rhizophagus*	HG004476.1	97%
OTU21	*Rhizophagus irregulare VTX00114*	*Glomerale*	*Glomeraceae*	*Rhizophagus*	JX144120.1	97%
OTU22	*Claroideoglomus* sp. *VTX00193*	*Glomerale*	*Claroideoglomeraceae*	*Claroideoglomus*	HQ258987.1	96%
OTU23	*Claroideoglomus* sp. *VTX00193*	*Glomerale*	*Claroideoglomeraceae*	*Claroideoglomus*	KJ809525.1	98%
OTU24	*Rhizophagus* sp.*VTX00113*	*Glomerale*	*Glomeraceae*	*Rhizophagus*	HG004521.1	99%
OTU25	*Claroideoglomus* sp.*VTX00057*	*Glomerale*	*Claroideoglomeraceae*	*Claroideoglomus*	KC708358.1	97%
OTU26	*Rhizophagus* sp.*VTX00113*	*Glomerale*	*Glomeraceae*	*Rhizophagus*	EU332711.1	96%
OTU27	*Claroideoglomus* sp. *VTX00193*	*Glomerale*	*Claroideoglomeraceae*	*Claroideoglomus*	KJ809525.1	98%
OTU28	*Claroideoglomus* sp. *VTX00193*	*Glomerale*	*Claroideoglomeraceae*	*Claroideoglomus*	HE615035.1	95%
OTU29	*Glomeraceae Glomus* sp. *VTX00114*	*Glomerale*	*Glomeraceae*	*Rhizophagus*	KC708370.1	99%
OTU30	*Claroideoglomus* sp. *VTX00193*	*Glomerale*	*Claroideoglomeraceae*	*Claroideoglomus*	HE614983.1	97%
OTU31	*Paraglomus* sp. *VTX00001*	*Paraglomerale*	*Paraglomeraceae*	*Paraglomus*	KF386326.1	89%
OTU32	*Rhizophagus irregulare VTX00114*	*Glomerale*	*Glomeraceae*	*Rhizophagus*	KC708370.1	99%
OTU33	*Glomeraceae* sp.*VTX00166*	*Glomerale*	*Glomeraceae*	-	FR693470.1	98%
OTU34	*Rhizophagus irregulare VTX00114*	*Glomerale*	*Glomeraceae*	*Rhizophagus*	KC708370.1	97%
OTU35	*Rhizophagus* sp.*VTX00113*	*Glomerale*	*Glomeraceae*	*Rhizophagus*	JX144125.1	96%
OTU36	*Acaulospora* sp. *VTX00028*	*Diversisporale*	*Acaulosporacea*	*Acaulospora*	EU332732.1	97%

Figure S1

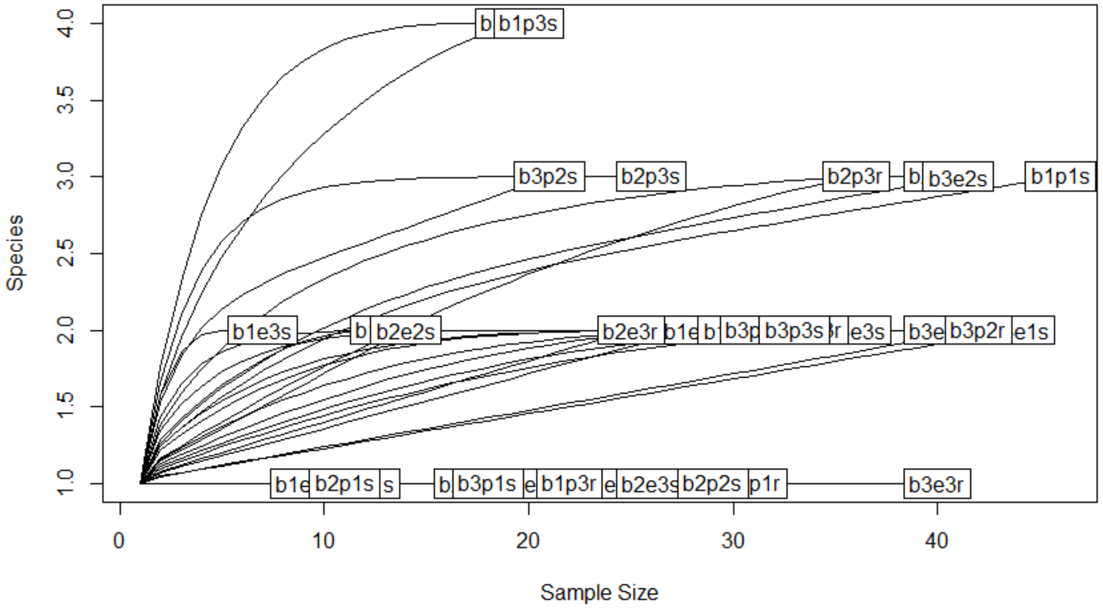

Figure S1. Rarefaction curves of each sample showing the saturation of OTUs richness of *Glomeromycota* associated with *P. tremuloides* and *E. elliptica* from low, moderate and high contamination basins. Rarefaction analysis was based on taxonomic assignment at 97% of sequence similarity.

Figure S2

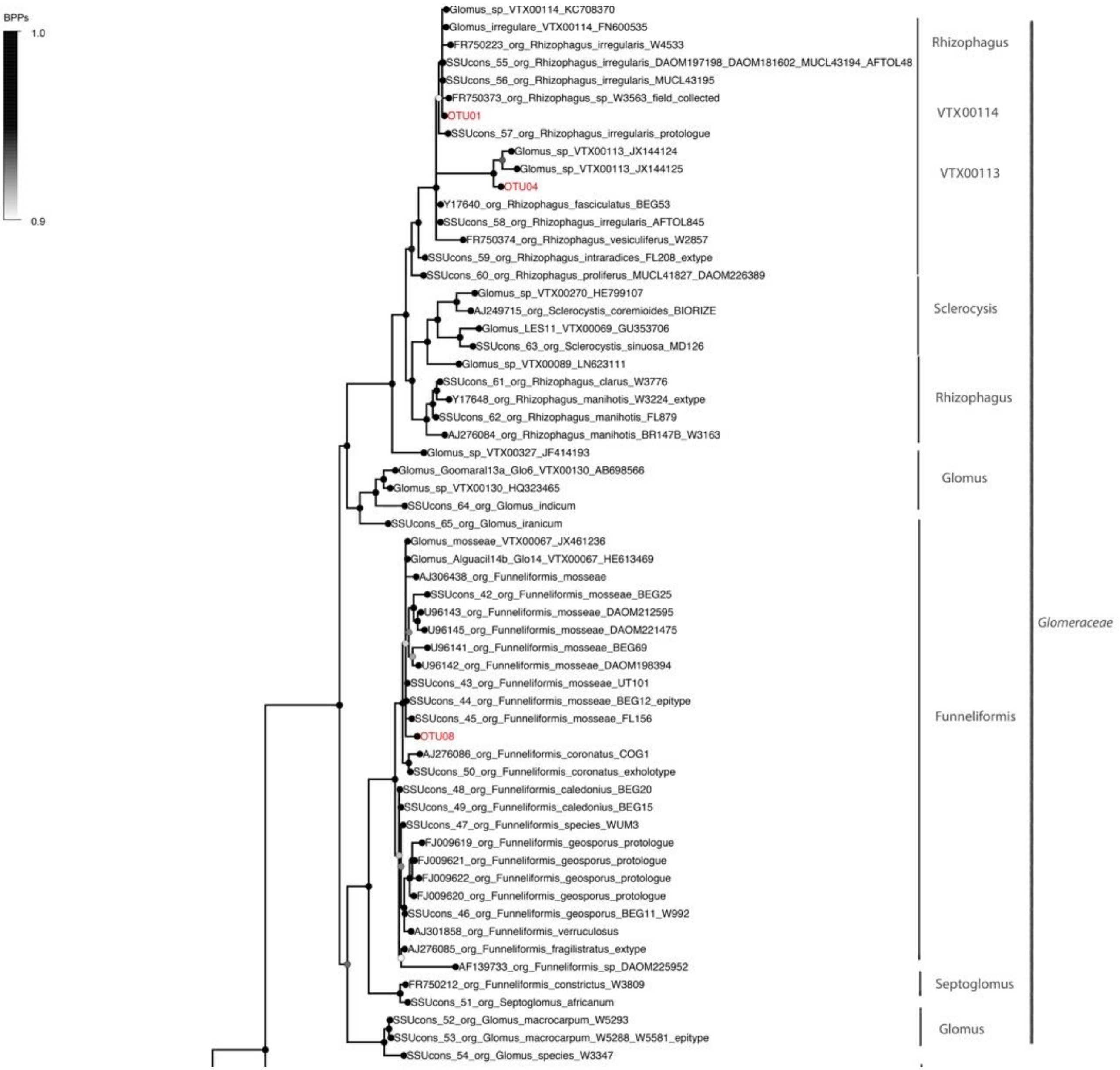

BPPs
1.0
0.9
Glomus_sp_VTX00114_KC708370
Glomus_irregulare_VTX00114_FN600535
FR750223_org_Rhizophagus_irregularis_W4533
SSUcons_55_org_Rhizophagus_irregularis_DAOM197198_DAOM181602_MUCL43194_AFTOL48
SSUcons_56_org_Rhizophagus_irregularis_MUCL43195
FR750373_org_Rhizophagus_sp_W3563_field_collected
OTU01
SSUcons_57_org_Rhizophagus_irregularis_protologue
Glomus_sp_VTX00113_JX144124
Glomus_sp_VTX00113_JX144125
OTU04
Y17640_org_Rhizophagus_fasciculatus_BEG53
SSUcons_58_org_Rhizophagus_irregularis_AFTOL845
FR750374_org_Rhizophagus_vesiculiferus_W2857
SSUcons_59_org_Rhizophagus_intraradices_FL208_extype
SSUcons_60_org_Rhizophagus_proliferus_MUCL41827_DAOM226389
Glomus_sp_VTX00270_HE799107
AJ249715_org_Sclerocystis_coremioides_BIORIZE
Glomus_LES11_VTX00069_GU353706
SSUcons_63_org_Sclerocystis_sinuosa_MD126
Glomus_sp_VTX00089_LN623111
SSUcons_61_org_Rhizophagus_clarus_W3776
Y17648_org_Rhizophagus_manihotis_W3224_extype
SSUcons_62_org_Rhizophagus_manihotis_FL879
AJ276084_org_Rhizophagus_manihotis_BR147B_W3163
Glomus_sp_VTX00327_JF414193
Glomus_Goomaral13a_Glo6_VTX00130_AB698566
Glomus_sp_VTX00130_HQ323465
SSUcons_64_org_Glomus_indicum
SSUcons_65_org_Glomus_iranicum
Glomus_mosseae_VTX00067_JX461236
Glomus_Alguacil14b_Glo14_VTX00067_HE613469
AJ306438_org_Funneliformis_mosseae
SSUcons_42_org_Funneliformis_mosseae_BEG25
U96143_org_Funneliformis_mosseae_DAOM212595
U96145_org_Funneliformis_mosseae_DAOM221475
U96141_org_Funneliformis_mosseae_BEG69
U96142_org_Funneliformis_mosseae_DAOM198394
SSUcons_43_org_Funneliformis_mosseae_UT101
SSUcons_44_org_Funneliformis_mosseae_BEG12_epitype
SSUcons_45_org_Funneliformis_mosseae_FL156
OTU08
AJ276086_org_Funneliformis_coronatus_COG1
SSUcons_50_org_Funneliformis_coronatus_exholotype
SSUcons_48_org_Funneliformis_caledonius_BEG20
SSUcons_49_org_Funneliformis_caledonius_BEG15
SSUcons_47_org_Funneliformis_species_WUM3
FJ009619_org_Funneliformis_geosporus_protologue
FJ009621_org_Funneliformis_geosporus_protologue
FJ009622_org_Funneliformis_geosporus_protologue
FJ009620_org_Funneliformis_geosporus_protologue
SSUcons_46_org_Funneliformis_geosporus_BEG11_W992
AJ301858_org_Funneliformis_verruculosus
AJ276085_org_Funneliformis_fragilistratus_extype
AF139733_org_Funneliformis_sp_DAOM225952
FR750212_org_Funneliformis_constrictus_W3809
SSUcons_51_org_Septoglomus_africanum
SSUcons_52_org_Glomus_macrocarpum_W5293
SSUcons_53_org_Glomus_macrocarpum_W5288_W5581_epitype
SSUcons_54_org_Glomus_species_W3347
Rhizophagus
VTX00114
VTX00113
Sclerocystis
Rhizophagus
Glomus
Funneliformis
Septoglomus
Glomus
Glomeraceae

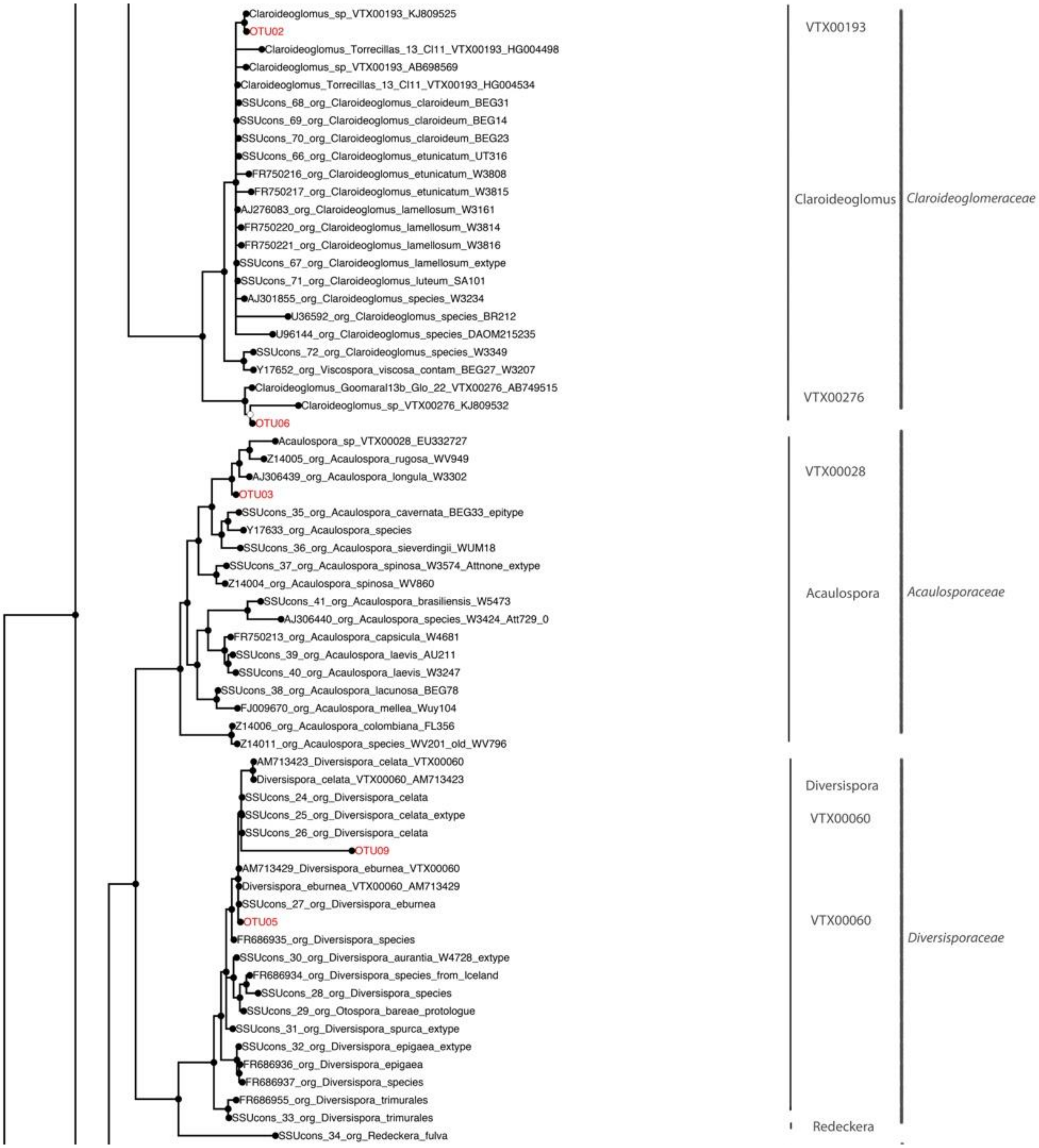
Claroideoglomus_sp_VTX00193_KJ809525
OTU02
Claroideoglomus_Torrecillas_13_Cl11_VTX00193_HG004498
Claroideoglomus_sp_VTX00193_AB698569
Claroideoglomus_Torrecillas_13_Cl11_VTX00193_HG004534
SSUcons_68_org_Claroideoglomus_claroideum_BEG31
SSUcons_69_org_Claroideoglomus_claroideum_BEG14
SSUcons_70_org_Claroideoglomus_claroideum_BEG23
SSUcons_66_org_Claroideoglomus_etunicatum_UT316
FR750216_org_Claroideoglomus_etunicatum_W3808
FR750217_org_Claroideoglomus_etunicatum_W3815
AJ276083_org_Claroideoglomus_lamellosum_W3161
FR750220_org_Claroideoglomus_lamellosum_W3814
FR750221_org_Claroideoglomus_lamellosum_W3816
SSUcons_67_org_Claroideoglomus_lamellosum_extype
SSUcons_71_org_Claroideoglomus_luteum_SA101
AJ301855_org_Claroideoglomus_species_W3234
U36592_org_Claroideoglomus_species_BR212
U96144_org_Claroideoglomus_species_DAOM215235
SSUcons_72_org_Claroideoglomus_species_W3349
Y17652_org_Viscospora_viscosa_contam_BEG27_W3207
Claroideoglomus_Goomaral13b_Glo_22_VTX00276_AB749515
Claroideoglomus_sp_VTX00276_KJ809532
OTU06
Acaulospora_sp_VTX00028_EU332727
Z14005_org_Acaulospora_rugosa_WV949
AJ306439_org_Acaulospora_longula_W3302
OTU03
SSUcons_35_org_Acaulospora_cavernata_BEG33_epitype
Y17633_org_Acaulospora_species
SSUcons_36_org_Acaulospora_sieverdingii_WUM18
SSUcons_37_org_Acaulospora_spinosa_W3574_Attnone_extype
Z14004_org_Acaulospora_spinosa_WV860
SSUcons_41_org_Acaulospora_brasiliensis_W5473
AJ306440_org_Acaulospora_species_W3424_Att729_0
FR750213_org_Acaulospora_capsicula_W4681
SSUcons_39_org_Acaulospora_laevis_AU211
SSUcons_40_org_Acaulospora_laevis_W3247
SSUcons_38_org_Acaulospora_lacunosa_BEG78
FJ009670_org_Acaulospora_mellea_Wuy104
Z14006_org_Acaulospora_colombiana_FL356
Z14011_org_Acaulospora_species_WV201_old_WV796
AM713423_Diversispora_celata_VTX00060
Diversispora_celata_VTX00060_AM713423
SSUcons_24_org_Diversispora_celata
SSUcons_25_org_Diversispora_celata_extype
SSUcons_26_org_Diversispora_celata
OTU09
AM713429_Diversispora_eburnea_VTX00060
Diversispora_eburnea_VTX00060_AM713429
SSUcons_27_org_Diversispora_eburnea
OTU05
FR686935_org_Diversispora_species
SSUcons_30_org_Diversispora_aurantia_W4728_extype
FR686934_org_Diversispora_species_from_Iceland
SSUcons_28_org_Diversispora_species
SSUcons_29_org_Otospora_bareae_protologue
SSUcons_31_org_Diversispora_spurca_extype
SSUcons_32_org_Diversispora_epigaea_extype
FR686936_org_Diversispora_epigaea
FR686937_org_Diversispora_species
FR686955_org_Diversispora_trimurales
SSUcons_33_org_Diversispora_trimurales
SSUcons_34_org_Redeckera_fulva
VTX00193
Claroideoglomus
Claroideoglomeraceae
VTX00276
VTX00028
Acaulospora
Acaulosporaceae
Diversispora
VTX00060
VTX00060
Diversisporaceae
Redeckera

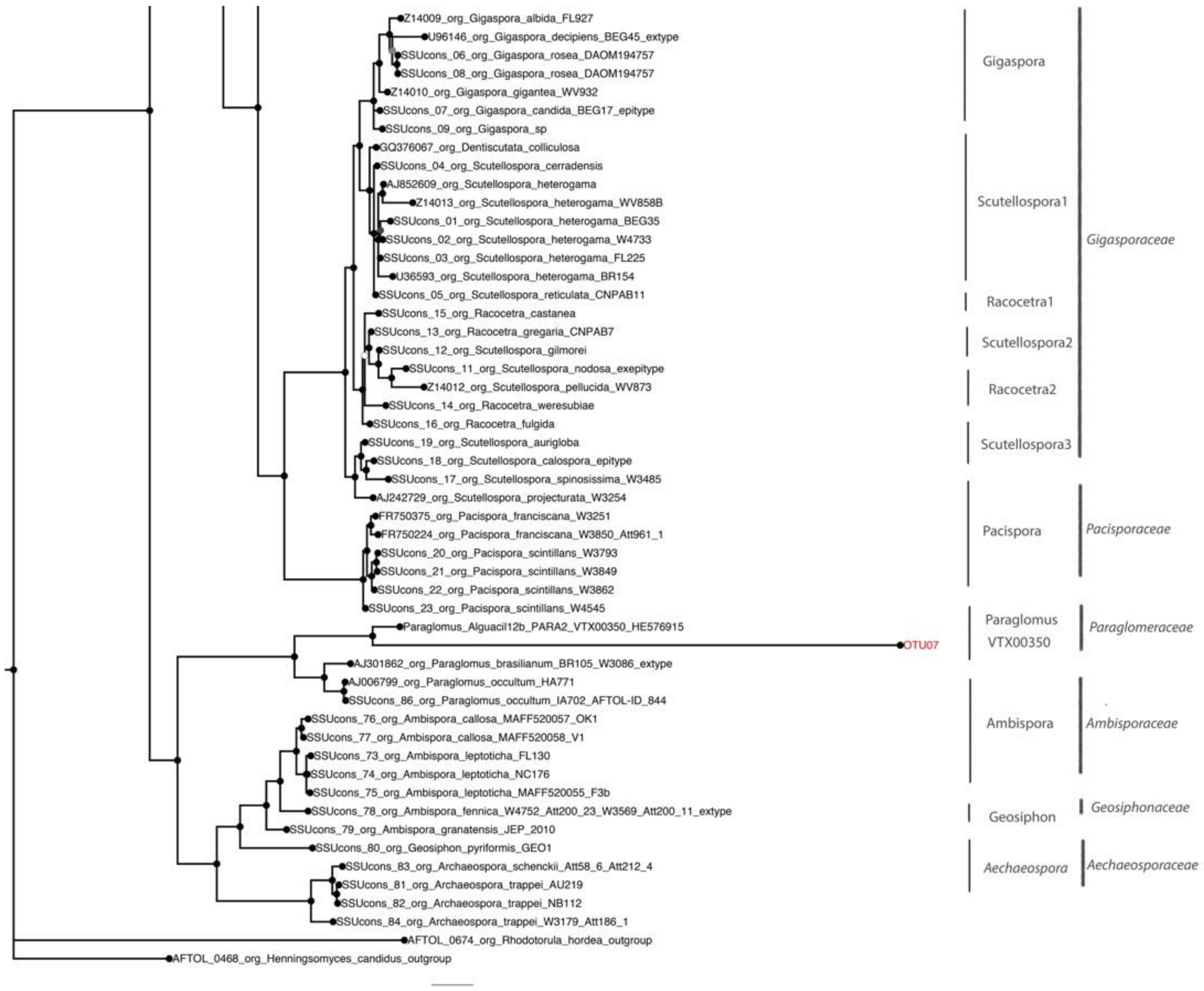

Figure S2. Bayesian phylogenetic tree based on nuclear small subunit (SSU) rDNA consensus sequences showing the distribution of the 9 OTUs recorded in contaminated sites (red labels) among the Glomeromycota phylogenetic tree. Sequence dataset was analyzed with the SSU sequences (black labels) from Krüger et al. (2012) and the closest match recovered from MaarjAM database. Circles on nodes indicate Bayesian posterior probabilities ranging from 0.9 (white) to 1 (black). The scale represents the branch length corresponding to expected substitutions per site.
